# Optimized Multiscale Entropy Model Based on Resting-State fMRI for Appraising Cognitive Performance in Healthy Elderly

**DOI:** 10.1155/2022/2484081

**Published:** 2022-06-07

**Authors:** Fan Yang, Fuyi Zhang, Abdelkader Nasreddine Belkacem, Chong Xie, Ying Wang, Shenghua Chen, Zekun Yang, Zibo Song, Manling Ge, Chao Chen

**Affiliations:** ^1^State Key Laboratory of Reliability and Intelligence of Electrical Equipment, Hebei University of Technology, Tianjin 300130, China; ^2^Hebei Province Key Laboratory of Electromagnetic Field and Electrical Apparatus Reliability, Hebei University of Technology, Tianjin 300130, China; ^3^Department of Computer and Network Engineering, College of Information Technology, United Arab Emirates University, Al Ain 15551, UAE; ^4^Key Laboratory of Complex System Control Theory and Application, Tianjin University of Technology, Tianjin 300384, China

## Abstract

Many studies have indicated that an entropy model can capture the dynamic characteristics of resting-state functional magnetic resonance imaging (rfMRI) signals. However, there are problems of subjectivity and lack of uniform standards in the selection of model parameters relying on experience when using the entropy model to analyze rfMRI. To address this issue, an optimized multiscale entropy (MSE) model was proposed to confirm the parameters objectively. All healthy elderly volunteers were divided into two groups, namely, excellent and poor, by the scores estimated through traditional scale tests before the rfMRI scan. The parameters of the MSE model were optimized with the help of sensitivity parameters such as receiver operating characteristic (ROC) and area under the ROC curve (AUC) in a comparison study between the two groups. The brain regions with significant differences in entropy values were considered biomarkers. Their entropy values were regarded as feature vectors to use as input for the probabilistic neural network in the classification of cognitive scores. Classification accuracy of 80.05% was obtained using machine learning. These results show that the optimized MSE model can accurately select the brain regions sensitive to cognitive performance and objectively select fixed parameters for MSE. This work was expected to provide the basis for entropy to test the cognitive scores of the healthy elderly.

## 1. Introduction

With the aging population becoming grim worldwide, the cognitive level of the old people has especially garnered massive attention [1–4] because it is related to the quality of life. Determining the cognitive ability of the elderly as early as possible is a prerequisite for prevention or intervention for slowing down the time course of degenerative or pathological cognitive decline. Therefore, the detection of cognitive ability of healthy old people is the basis for distinguishing between a degenerative and pathological decline, and it is also an effectual control to evaluate cognitive impairment [5–7].

The brain is a complex nonlinear system, and it is thus essential to study the complexity of physiological signals from the perspective of nonlinear dynamics. Physiological complexity can reflect functional changes by quantitatively analyzing the regularity (orderliness) in the time-series signals of the brain [8–10]. Studies have evidenced that brain nerve cells also have cooperative activities in the resting state (closed eyes, awake, no specific cognitive task) and maintain the complex network system that appears in the task state. The low-frequency fluctuation of the blood-oxygen-level-dependent (BOLD) signal is not random noise. It can reflect the spontaneous neural activity of the human brain, which has certain physiological significance [11, 12]. According to studies, the BOLD signals measured by resting-state functional magnetic resonance imaging (rfMRI) are important for modeling the link between blood flow and neural activity, which is an advanced tool for us to learn about the human brain activity [13]. rfMRI not only has the advantages of high spatial resolution and noninvasiveness [14] but is also faster (less than 15 min) than task-state fMRI [13, 15]. In recent decades, rfMRI as a novel technique offers a remarkable opportunity to explore cognitive studies. Khazaee et al. used rfMRI to study functional brain network alterations in patients with Alzheimer's disease (AD) [16]. Hojjati et al. predicted conversion from mild cognitive impairment to AD by integrating rfMRI and structural MRI [17].

Entropy, as a classical physical parameter, is extensively used in nonlinear systems, such as medicine [18], electricity [19], machinery [20], and other sciences and humanities fields [21]. The entropy model has gone through the development process of approximate entropy (ApEn), sample entropy (SampEn), multiscale entropy (MSE), etc. The ApEn model, first proposed by Pincus, can estimate the complexity of time series from a small amount of data. One of its advantages is classifying complex systems into two categories, namely, deterministic and stochastic [22]. The SampEn model, improved by Richman, was eventually declared the solution to the probability deviation caused by the self-matching problem of the ApEn model, and it could be simpler than the ApEn model and less dependent on the length of the time series [23]. However, the model was difficult to adapt to different states in a complex system because of its fixed scale. Therefore, the concept of MSE was proposed to eliminate the error caused by a fixed scale. Generally, SampEn quantifies the temporal irregularity of temporal patterns in the signal. As an extension of SampEn, MSE aimed to describe temporal irregularity at different time scales—varying from fine to coarse [24].

MSE has an adequate recognition ability for biomedical signals without the self-matching defect in calculating probability. The MSE values derived from the heart rate variability can clarify the difference between healthy and diseased states, substantiating the theory of “complexity loss” of aging and disease that with the aging or disease of the human body, and the complexity of physiological signals would gradually decrease [1, 25, 26]. Furthermore, MSE values derived from biomedical signals can also assess different physiopathological states [27–29]. Turianikova et al. found that MSE analysis of heart rate and blood pressure is sensitive to changes in autonomic balance [29]. Blons et al. found that changes in cardiac entropy accompany acute responses to cognitive load and stress [27].

By dint of fMRI, the MSE model can uncover changes in brain physiology, pathology, and functions from the complexity perspective [30–32]. Therefore, applying the MSE model to study the complexity of rfMRI may provide new ideas for evaluating the cognitive scores of the healthy elderly.

Conventionally, there are three ways to select the optimal parameters for complexity measures of BOLD signals. The first one is to roughly select empirical values for parameters according to previous work and signal features. The second one is to select a range of parameters that maximize the significant differences in complexity measures of brain BOLD signals between two different groups. The third one is to minimize the relative error of the entropy of BOLD signal in cerebrospinal fluids that could contain minimal physiologic information but uncorrelated noise [33, 34]. Ran et al. developed a novel *K*-means clustering algorithm to automatically select the optimal number of clusters and sensitively initialize the center cluster [35]. Cui et al. adaptively optimized and determined the system parameters of stochastic resonance by using the subsampling technique [36]. Taking the evaluation of the cognitive performance of the healthy elderly as an example, this paper tried to solve the problem that MSE parameter selection depended on the experience and lacked a unified objective basis [37].

This paper highlighted the importance of optimizing parameters in the MSE model and innovatively optimized the parameters of multiscale entropy through the receiver operating characteristic (ROC) and area under the ROC curve (AUC). The optimized MSE model could accurately extract the functional imaging markers sensitive to the cognitive scores. This study integrated the optimized MSE model with a machine learning model to classify the cognitive scores of the healthy elderly.

## 2. Materials and Methods

**Figure 1:**
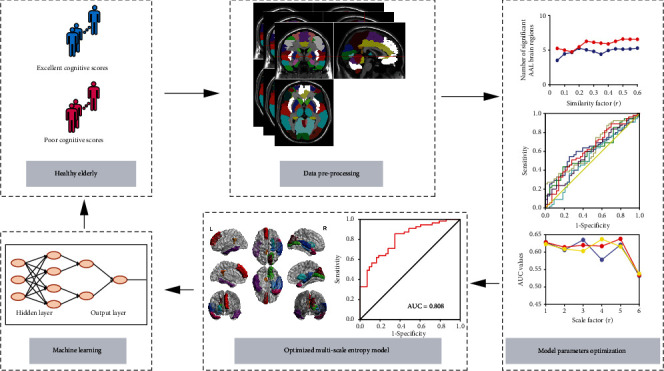
Study flowchart.

The process of this study can generally be divided into the following steps: (1) the MSE model parameters were optimized by the “maximizing between-group difference” approach. Using the ROC curve and AUC values, the approach can find a combination of parameters that maximize the differences of BOLD complexity between two groups. (2) The functional biomarkers of brain regions sensitive to the cognitive scores were validated by the statistical significance of the optimized entropy values between two groups. (3) The optimized entropy values of biomarker areas were regarded as feature vectors input into the probabilistic neural network (PNN) to classify the cognitive scores. The accuracy of classification was tested by *N*-fold crossvalidation. This study's flowchart is depicted in Figure 1.

### 2.1. Subjects

The elderly participants were selected from a public dataset of a cohort study involving 1,051 Portuguese elderly people over 50 years old (http://github.com/juanitacabral/LEiDA) whose cognitive behaviors were assessed by the scores derived from a total of nine series of neuro-psychological scale tests that they conducted before the rfMRI scan.

Principal component analysis was used to determine the scores of two main dimensions related to memory and cognitive executive functions: Mini-Mental State Examination (MMSE) and Geriatric Depression Scale (GDS, long version). These two dimensions scores did not form any correlation with other grouping of neuropsychological variables. Based on the scores of these two dimensions, four separate cluster solutions were tested, ranging from 2 to 5. To determine the most suitable solution, an analysis of variance was performed on each cluster solution, and the four-cluster solution was deemed to provide the best cluster solution. According to cognitive performance, 1051 subjects were ranked C1 > C2 > C3 > C4, where C1 and C4 represented an excellent cognitive level and a poor cognitive level, respectively [38].

Then, two groups of participants were formed by randomly selecting 60 subjects from each of the above C1 and C4 groups resulting in two groups with different cognitive performance: 60 subjects with excellent cognitive performance and 60 subjects with poor cognitive performance. In the subsample of 120 subjects, 9 subjects refused to undergo MRI screening, 4 had previously undiagnosed brain pathologies, and 9 subjects were excluded due to excessive motion. The final samples consisted of 98 subjects, 55 with excellent cognitive scores (denoted as the excellent group) and 43 with the lowest cognitive scores (denoted as the poor group) [39, 40].

### 2.2. Data Acquisition

During the scanning process, participants were asked to keep their eyes closed, stay awake, and not think of anything in particular. Data of rfMRI were collected at Braga hospital, Portugal, by using a clinically approved 1.5 T Siemens magnet Avanto 12-channel head coil scanner. Using BOLD-sensitive echo plane imaging sequence, the parameters were set as follows: 30 axial slices, TR/TE = 2000/30 ms, FA = 90°, slice gap = 0.48 mm, and voxel size = 3.5 × 3.5 × 3.5 mm^3^.

This study was performed in accordance with the Declaration of Helsinki (59th amendment), and all subjects provided written informed consent.

### 2.3. Data Preprocessing

The preprocessing of rfMRI data was performed using FMRIB software library tools [41, 42]. The steps followed can be enumerated as follows: (1) the first five data values were removed to stabilize the signal; (2) slice timing was corrected; (3) MCFLIRT [41] software was used to align each volume of a rigid body with the average image for motion correction (FD < 0.2 mm); (4) brain extraction tool was used for skull peeling [43]; (5) FLIRT software was used to obtain the structure through continuous rigid body registration to normalize the nonlinear function. The nonlinear registration was due to the original space of the structure to the Montreal neurological institute standard space, and FNIRT was used to resample to a 2 mm^3^ isotropic voxel size; (6) linear regression of motion parameters, average cerebral spinal fluid and white matter signals, and other customized covariables, such as age or gender of subjects; and (7) band-pass time filtering of regression residual (0.01–0.08 Hz). To compute the MSE, the mean time course was extracted for each brain region by the anatomical automatic labeling (AAL) atlas.

### 2.4. Sample Entropy

For one-dimensional *N*-length discrete time series {*x*_1_, *x*_2_, *x*_3_, ⋯, *x*_*N*_}, a new coarse-grained time series {*y*(*τ*)} is obtained by transforming Equation (1):

(1) $$\begin{array}{c} y_{j}^{\tau }=\frac{1}{\tau }\overset{j\tau }{\underset{i=\left(j-1\right)\tau +1}{\sum }}x_{i}. \end{array}$$

**Figure 2:**
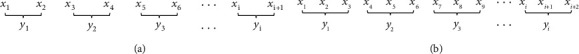
Coarse-graining procedure. (a) Scale factor 2. (b) Scale factor 3. *x* is the original time series, and *y* is coarse-grained time series.

A coarse-graining procedure at scale factors 2 and 3 is illustrated in Figure 2.

1 ≤ *j* ≤ *N*/*τ*, *τ*, is the scale factor, and the length of {*y*(*τ*)} is *L* = *N*/*τ*.

Then, a set of *m*-dimension vectors (*m* is embedding dimension) *Y*_*m*_ (*i*) that is formed: *Y*_*m*_(*i*) = *y*_*i*+*k*_, {0 ≤ *k* ≤ *m* − 1}. For each *i* value, calculate its distance from other *j* values, that is, the distance between *Y*_*m*_ (*i*) and *Y*_*m*_ (*j*), shown in Equation (2):

(2) $$\begin{array}{c} d\left[Y_{m}\left(i\right),Y_{m}\left(j\right)\right]=\max\left|y\left(i+k\right)-y\left(j+k\right)\right|\;\left(0\leq k\leq m-1,i,j=1-L-m+1,i\neq j\right). \end{array}$$

Setting the tolerance threshold (i.e., similarity factor) *r* (*r* > 0), the number *B*_*m*_ (*i*) of *d* [*Y*_*m*_(*i*), *Y*_*m*_(*j*)] < *r* is calculated for each *i* value, and its ratio with the total distance can be obtained using Equation (3):

(3) $$\begin{array}{c} C_{\tau }^{m}\left(r\right)=\frac{B^{m}\left(i\right)}{L-m}. \end{array}$$

Then, the average of *C*_*τ*_^*m*^(*r*) can be found using Equation (4):

(4) $$\begin{array}{c} C^{m}\left(r\right)=\frac{1}{L-m+1}\overset{L-m+1}{\underset{i=1}{\sum }}C_{\tau }^{m}\left(r\right). \end{array}$$

Similarly, the *m* + 1 dimension can be derived using Equation (5):

(5) $$\begin{array}{c} C^{m+1}\left(r\right)=\frac{1}{L-m}\overset{L-m}{\underset{i=1}{\sum }}C_{\tau }^{m+1}\left(r\right). \end{array}$$

When *L* is a finite value, the SampEn with the sequence length *L* can be estimated as SampEn in Equation (6):

(6) $$\begin{array}{c} \text{SampEn}\left(m,r\right)=-\ln\left[\frac{C^{m+1}\left(r\right)}{C^{m}\left(r\right)}\right]. \end{array}$$

A high entropy value indicates a lot of complexity in the time course and vice versa.

### 2.5. MSE

With a total of three parameters to estimate MSE, namely, the scale factor  *τ*, embedding dimension *m*, and similarity factor *r*, a set of SampEn values will be formed, denoted as MSE in Equation (7):

(7) $$\begin{array}{c} \text{MSE}=\left\{\tau \left|\text{SampEn }\left(m,r\right)=-\ln\left[\frac{C^{m+1}\left(r\right)}{C^{m}\left(r\right)}\right]\right|\right\}. \end{array}$$

When computing the MSE value, a very short length of time series data will make the SampEn unreliable. According to Richman and similar studies, when calculating the SampEn by BOLD time series, the length of 10^m^–20^m^ data should be enough to estimate the SampEn [22]. For a short-term BOLD signal processing, at least 10–20 data are needed when *m* = 1, and 100-400 data values are required when *m* = 2.

### 2.6. Optimization Parameters

The selection of the MSE model parameters such as dimension *m* and similarity *r* is dependent on the signal features and is determined by the experience. Based on data length and previous work, the empirical value for parameters could be roughly selected [34]. For example, Protzner et al. chosen parameters *m* = 2 and *r* = 0.5 when the time series length of EEG was 400 points [44]; the length of the time series was 40,000, and *m* = 2 and *r* = 0.15 were employed [45]. Costa selected *m* = 2 and *r* = 0.15 when time series with 3000 points in a study of heart rate [8]. In other words, there is an absence of a uniform parameter standard or specification for MSE to process biomedical signals, which would lead to subjectivity in entropy calculation.

Because of the considerations above, it is necessary to optimize the parameters of the MSE model. Herein, an approach is proposed that the sensitivity indexes, namely, the ROC curve and AUC values, are employed to indicate the parametric optimization effects quantitatively. The ROC curve can directly display the classification effect, and the AUC values can quantitatively show the optimization effect [46].

The higher the ROC curve above the reference line and the greater the AUC values, the better the optimization effect is, and vice versa. Customarily, an AUC value could be divided into five levels, i.e., 0.50–0.59, 0.60–0.69, 0.70–0.79, 0.80–0.89, and 0.90–1, representing poor, bad, medium, good, and excellent classification effect, respectively [46].

Considering the mutual influence of the parameters in the MSE model, all three parameters are integrated to assess the optimization effect during the optimization process of each parameter.

### 2.7. Functional Biomarker Brain Regions

The brain regions with the significant difference in the AUC values between the two groups could be considered the functional biomarker sensitive to the cognitive scores while optimizing the parameters. Otherwise, the brain region cannot be regarded as a functional biomarker. All 90 cortical brain regions were involved in assessing functional biomarkers of a person. The *p* values of the *t*-test revealed the significance using the Statistical Package for the Social Sciences (SPSS) software (IBM SPSS statistics 21; USA). The significance level of this paper is set to 0.05. Finally, using BrainNet Viewer visualizes the marked brain regions sensitive to the scores of elders (http://www.nitrc.org/projects/bnv/) [47].

### 2.8. PNN Model and *N*-Fold Crossvalidation

After the functional biomarkers were confirmed, the optimized entropy values of those brain regions were averaged over the subjects. The average entropy values were regarded as feature vector input into the machine learning model. The elderly with excellent cognitive scores were marked as “1,” and those with poor cognitive scores were marked as “0.”

**Figure 3:**
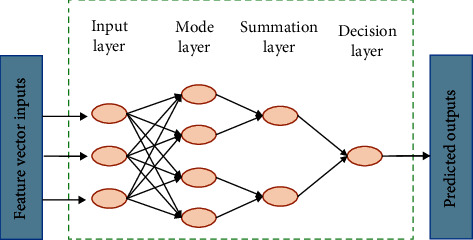
Structure of PNN.

The machine learning model of PNN was employed to classify the cognitive scores. A PNN is a nonlinear model used for classification and prediction by estimating conditional probability [48]. It is a forward neural network model derived from a radial basis neural network by combining a density function estimation with Bayesian decision theory. This model replaces the *S*-type function commonly used in neural networks with an exponential function as the activation function. This neural network model based on the statistical principle does not need the connection weights of training samples and directly constitutes the hidden layer from given samples. The training is simple, and the classification ability is strong. The PNN model consists of four layers: input, mode, summation, and decision, as shown in Figure 3.

As a training set, 34 subjects were randomly selected from 43 subjects in the poor group. The rest nine subjects were included in the test set. Likewise, from 55 subjects in the excellent group, 44 subjects were randomly selected for training set, and the remainder 11 subjects were included in the test set. Thus, 78 subjects formed the training set, and 20 subjects formed the test set.

In the PNN model, 98 neurons in the input layer and 20 neurons in the summation layer were selected at first. The expected classes were then transformed into vectors. We changed the smoothing factor from 0.1 to 2 in steps of 0.1, and the classifier had the highest accuracy when the smoothing factor was 1.5. The smoothing factor parameter was set as 1.5 for the network prediction. The classification effects on the training data and the prediction effect on the unknown data were observed through mapping. To appraise the classification accuracy, it was noted whether the prediction outputs were consistent with the known original outputs.

*N*-fold crossvalidation is often used to appraise the algorithm accuracy of classification in a machine learning model. In a small scale of samples, the dataset is divided into *N* parts, where *N* − 1 parts are regarded as training data and 1 part as test data in turn for the test procedure. An accuracy (or error rate) can be obtained in each test. Thus, the accuracy of classification algorithm can be determined by averaging the error rate over *N* tests. In this study, the value of *N* was taken to be 10.

## 3. Results

### 3.1. Parametric Optimization of MSE Model

#### 3.1.1. Optimization of Embedding Dimension *m*

After preprocessing, the length of the BOLD signal was observed to be 175 time points in the elderly. Thus, the value of *m* can be 1 or 2. Therefore, according to previous experience (see MSE in Section 2.5), the optimized parametric spaces were initially confirmed in a range of *m* = 1 − 2, *r* = 0.05 − 0.60, and  *τ* = 1 − 6.

**Figure 4:**
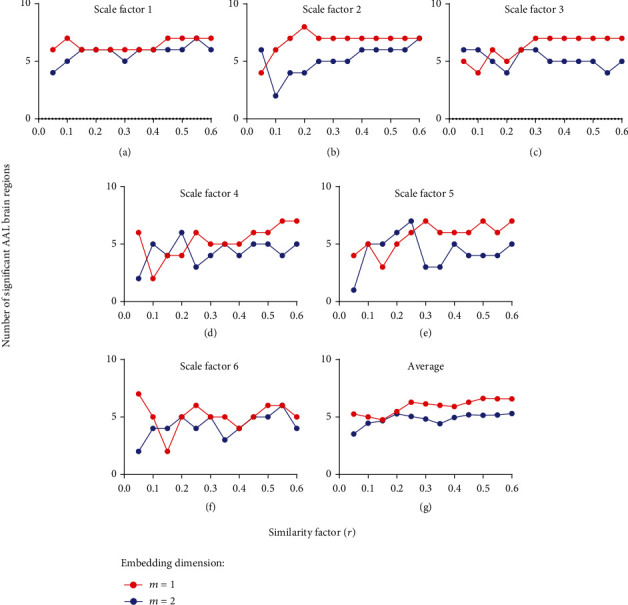
Optimization of embedding dimension m by the number of significant brain regions: (a) *τ* = 1, (b) *τ* = 2, (c) *τ* = 3, (d) *τ* = 4, (e) *τ* = 5, (f) *τ* = 6, and (g) average number of significant brain regions over the scale factor *τ*.

After the *t*-test, the embedding dimension *m* was optimized by the number of significant brain regions between two groups (*p* < 0.05), with the scale factor *τ* = 1 − 6, similarity factor  *r* = 0.05–0.6, and step size is 0.05, as shown in Figure 4. It was found that the number of significant brain regions of *m* = 1 was greater than that of *m* = 2, suggesting that the former was better than the latter. Thus, *m* = 1 was the optimization parameter. Additionally, only a very narrow value space of the similarity factor *r*, e.g., *r* = 0.45–0.55  could show significant differences on all scale factor values *τ*.

#### 3.1.2. Optimization of the Similarity Factor *r* and Scale Factor *τ*

**Figure 5:**
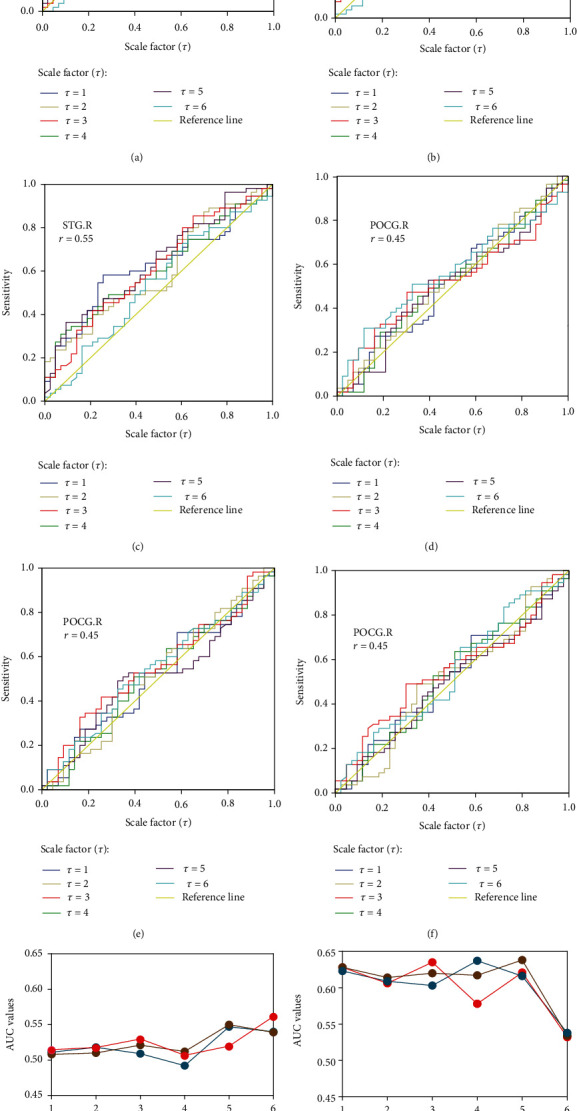
Plot optimization effects indicated by ROC curves and AUC values in a single brain region. (a)–(c) ROC curves of STG.R with *r* = 0.45, 0.50, and 0.55, respectively, where the character of all ROC curves beyond the reference lines indicates STG.R to be a functional biomarker. (d)–(f) ROC curves of PoCG.R with *r* = 0.45, 0.50, and 0.55, respectively, where ROC curves around the reference lines suggest that PoCG.R was not a functional biomarker. (g) AUC values of STG.R. (h) AUC values of PoCG.R.

Taking the brain regions of the right superior temporal gyrus (STG.R) and right postcentral gyrus (PoCG.R) as examples, the optimization of similarity factor *r* and scale factor  *τ*  was shown by the features of ROC curves and AUC values in Figure 5 and Table 1.

By setting *m* = 1 fixed and *r* = 0.45, 0.50, and 0.55, respectively, the ROC curves of STG.R at different *τ* values are shown in Figures 5(a)–5(c), and it can be seen that the ROC curves were all above the reference lines. So, STG.R could be considered sensitive to the cognitive scores. In contrast, the ROC curves around the reference lines displayed that the PoCG.R cannot be a functional biomarker in Figures 5(d)–5(f).

Furthermore, Figures 5(g) and 5(h) show that the AUC values of STG.R were greater than those of PoCG.R, and they were largest at *τ* = 5, suggesting that *τ* = 5 was the optimized value. In particular, when  *τ* = 5, the AUC value of STG.R was the largest at *r* = 0.5 as highlighted in Table 1, indicating that *r* = 0.5 was the optimized value.

The same optimization method was applied to other brain regions. Similar features and optimized parameters of *τ* = 5 and *r* = 0.50 were found. Taken together, the optimized parameters of entropy model were *m* = 1, *r* = 0.5, and  *τ* = 5.

#### 3.1.3. Functional Biomarker Brain Region and Feature Vector Extraction

**Figure 6:**
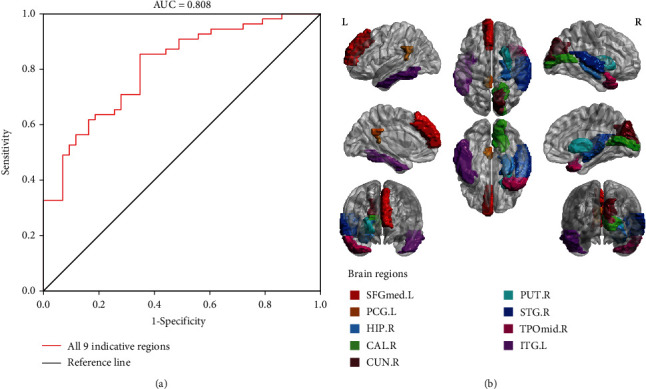
(a) ROC curve and AUC value of a total of nine functional biomarked brain regions at the optimization parameters of *m* = 1, *r* = 0.5, and *τ* = 5 in the MSE model. (b) Landmark brain regions shown on a brain template using BrainNet Viewer.

With the optimized MSE values of *m* = 1, *r* = 0.5, and *τ* = 5, a total of nine AAL brain regions sensitive to cognitive scores were obtained (*p* < 0.05), i.e., right calcarine fissure and surrounding cortex (CAL.R, AAL44), left medial superior frontal gyrus (SFGmed.L, AAL23), left posterior cingulate gyrus (PCG.L, AAL35), left inferior temporal gyrus (ITG. L, AAL89), right superior temporal gyrus (STG.R, AAL82), right cuneus (CUN.R, AAL46), right lenticular nucleus, putamen (PUT.R, AAL74), right hippocampus (HIP. R, AAL38), and right temporal pole: middle temporal gyrus (TPOmid.R, AAL88); all were located in the default mode network (DMN) and surrounding areas. The classification effect of the nine landmark brain regions and their projections on the cortical surface is depicted in Figure 6.

The matrix of feature vector was formed after averaging the entropy values over all nine functional biomarkers. The training set data and the testing set data were then built up. In Tables 2 and 3, when *m* = 1, *r* = 0.5, and *τ* = 5, there was a significant difference between the excellent group and the poor group (*p* < 0.001), supporting the optimization method proposed above.

### 3.2. Classification by PNN and *N*-Fold Crossvalidation

The classification accuracy of the PNN reached 80%. Then, *N*-fold crossvalidation was carried out (*N* = 10), the max classification accuracy was 88.24%, and the average accuracy was 80.05% (see Table 4).

## 4. Discussion

### 4.1. Parametric Optimization of MSE Model

MSE implementations use low-pass filters to coarse-grain the original signal at coarser time scales, which introduces a sensitivity to slower neural dynamics. This is related to the scale factor *τ*, and the higher *τ* means the slower neural dynamics [24]. In the past, there was no uniform standard or specification for entropy parameters of *r* and *m* to process biomedical signals. By the rfMRI signal characteristics, a parametric optimization of the MSE model was conducted with the help of classification effect indexes. This optimization overcame the blindness and subjectivity in the calculation of MSE and enhanced the objectivity of parameter selection. Koltcov et al. proposed an approach based on Renyi entropy to solve the issue of parameter optimization in hierarchical models [49]. Siuly et al. investigated permutation entropy and autoregressive model features to explore changes in EEG signals that effectively differentiate mild cognitive impairment (MCI) from healthy control subjects [50]. Our means supported classifying early MCI and late MCI using the ROC curve and AUC values.

To test the generalizability of the MSE model, an additional experiment was performed, which included 112 subjects (56 major depressive disorder (MDD) and 56 healthy individuals as a control), as detailed in the Supplementary Material Section 1. According to Figure S1, the embedding dimension *m* can be optimized, and the optimization of similarity factor *r* and scale factor *τ* was shown by the features of ROC curves and AUC values in Figure S2 and Table S1 in Supplementary Materials. With the optimized MSE values of *m* = 1, *r* = 0.54, and *τ* = 3, nine AAL brain regions sensitive to MDD were obtained, including the left amygdala (AMYG.L, AAL41), left inferior parietal (IPL.L, AAL61), and left precuneus (PCUN.L, AAL67), among others (see Supplementary Figure S3). Additionally, significant differences in 9 brain regions between MDD and healthy individuals are shown in Supplementary Table S2. Finally, the classification accuracy of the PNN reached 75.83%, as detailed in Supplementary Table S3.

### 4.2. MSE and Conventional FC Model of Pearson Correlation

The critical technology of evaluating the cognitive scores by entropy was to find the functional biomarked brain regions that were sensitive to the cognitive scores (here, the cognitive scores were referred by a series of scale tests before the rfMRI scan). Our findings of the functional biomarkers on the DMN highly converged with previous work through the functional connection (FC) calculated using Pearson correlation coefficient. Yang et al. found that the MSE values of BOLD signals on DMN were positively correlated with the main cognitive functions, such as attention, orientation, short-term memory, mental control, and language [51]. Some studies have shown that the correlation between MSE and the functional connection calculated by the Pearson correlation coefficient depends on the dynamic character of BOLD signals such as frequency [31]. However, when the optimized MSE model was replaced with the conventional FC model of Pearson correlation in this study, as detailed in the Supplementary Material Section 2, the mean classification accuracy was only 60.33% (see Supplementary Table S4).

In addition, our work of functional biomarker brain regions was fairly congruent with the evidence concerning brain structure. Wang et al. found that there were significant changes in the mediating centrality of the right anterior cingulate gyrus, crevicular fissure and peripheral cortex, lenticular putamen, and left anterior cingulate gyrus of patients with amnesic mild cognitive impairment (aMCI) [52]. Moreover, Smart et al. found that the brain structure in subjective cognitive decline was thinner than that in the middle area of right tail, left posterior central gyrus, right cuneiform lobe, right paracentral lobule, right calcarine fissure, surrounding area, right middle frontal area, and right temporal polar cortex [53].

### 4.3. Other Different Machine Learning Models

Other different types of machine learning were tested, such as support vector machine (SVM), random forest (RF), and *K*-nearest neighbors (KNN), and 10-fold crossvalidation was carried out, as detailed in the Supplementary Material Section 3. The performance of different machine learning modes was compared. The average accuracy of SVM, RF, and KNN is 74.60%, 65.84%, and 71.47%, respectively (see Supplementary Table S5). Finally, the PNN used in this paper has the best results in classifying cognitive scores. The results obtained by different machine learning models further demonstrated the effectiveness of the optimized MSE model.

### 4.4. Sensitivity of MSE

Some studies have demonstrated the sensitivity of MSE of rfMRI. Niu et al. analyzed the MSE of four groups of subjects, including healthy subjects and patients with early and late MCI and AD and as a control group by a statistics of one-way analysis of variance [54]. It was found that there were significant differences in several scale factors among thalamus, insula, lingual and suboccipital gyrus, superior frontal gyrus and olfactory cortex, superior marginal gyrus, superior temporal gyrus, and middle temporal gyrus [55, 56]. Compared to the healthy group, the complexity of the BOLD signal in patients with MCI and AD was significantly reduced, while the complexity in AD patients was lower than that in MCI patients. This is the first study to appraise the sensitivity of MSE of rfMRI to the cognitive scores in the healthy elderly.

### 4.5. Limitations and Prospects

It is unknown whether the entropy model parameters can classify the cognitive scores at the medium level yet, which needs to be examined further by acquiring pertinent data. In addition, the brain structure of all the subjects in this paper had not changed significantly. How to optimize the parameters of the entropy model in accordance with the changes in the brain structure of the elderly can be studied in the future. Furthermore, we divided the brain into 90 brain regions by the AAL atlas, which is coarse. Applying templates that more finely divided brain regions, such as Yeo 400 atlas and Power 256 atlas, would reveal more brain regions sensitive to cognition.

## 5. Conclusion

This paper finds out the optimized parameters of the MSE model by the ROC and AUC values. Using an optimized MSE model, a total of nine landmarks sensitive to the cognitive scores of the healthy elderly could be obtained in right calcarine fissure and surrounding cortex (CAL.R), left medial superior frontal gyrus (SFGmed.L), left posterior cingulate gyrus (PCG.L), left inferior temporal gyrus (ITG. L), right superior temporal gyrus (STG.R), right cuneus (CUN.R), right lenticular nucleus, putamen (PUT.R), right hippocampus (HIP.R), and right temporal pole: middle temporal gyrus (TPOmid.R). The MSE values of these brain regions were input into a machine learning model as feature vectors, and 80.05% classification accuracy was obtained. The result shows that the parameters of MSE can be optimized objectively through ROC and AUC values. In addition, the MSE value is closely related to cognitive behavior and can effectively distinguish the cognitive performance of the healthy elderly.

## Figures and Tables

**Table 1:** Effect of similarity factor *r* and scale factor *τ* on sorting rate by AUC value of each brain region.

*τ*	*r*					
	0.45		0.50		0.55	
	STG.R	PoCG.R	STG.R	PoCG.R	STG.R	PoCG.R
1	0.628	0.514	0.628	0.508	0.623	0.511
2	0.606	0.517	0.614	0.510	0.609	0.518
3	0.635	0.529	0.620	0.521	0.603	0.509
4	0.578	0.506	0.617	0.512	0.637	0.492
5	0.621	0.519	0.683	0.550	0.616	0.547
6	0.532	0.561	0.534	0.539	0.538	0.540

**Table 2:** Between-group difference significance of feature vectors for different similarity factors (*r*).

*r*	Significance (*p* value)
0.15	0.6220
0.25	0.0358
0.35	0.0160
0.45	0.0027
0.50	<0.001

**Table 3:** Between-group difference significance of feature vectors for different scale factors (*τ*).

*τ*	Significance (*p* value)
1	0.0559
2	0.0328
3	0.0069
4	0.0101
5	<0.001

**Table 4:** Classification rate (CR) tested by 10-fold crossvalidation.

*N*	CR (%)	*N*	CR (%)	*N*	CR (%)
1	88.24	6	81.82		
2	70.59	7	88.24		
3	81.82	8	68.95	Average ± std	80.05 ± 7.82
4	68.95	9	88.24		
5	81.82	10	81.82		

## Data Availability

The participants in this experiment were taken from a public data set (http://github.com/juanitacabral/LEiDA).

## Supplementary Materials

Supplementary Materials An additional experiment of MDD was performed in Supplementary Material Section 1 to improve the generalizability of the optimized MSE model. The FC model of Pearson correlation was used to classify the cognitive scores of the healthy elderly, whose methodological description and results were depicted in Supplementary Material Section 2. In Supplementary Material Section 3, other three popular machine learning models were employed to validate the classification performance of the optimized MSE model.
