# Supplementary material for: Optimized Multiscale Entropy Model Based on Resting-State fMRI for Appraising Cognitive Performance in Healthy Elderly
Source: Comput Math Methods Med. 2022 Jun 7;2022:2484081. doi: 10.1155/2022/2484081 (PMC9197667; doi:10.1155/2022/2484081)
Supplement: Supplementary Materials — An additional experiment of MDD was performed in Supplementary Material Section 1 to improve the generalizability of the optimized MSE model. The FC model of Pearson correlation was used to classify the cognitive scores of the healthy elderly, whose methodological description and results were depicted in Supplementary Material Section 2. In Supplementary Material Section 3, other three popular machine learning models were employed to validate the classification performance of the optimized MSE model. [file 2484081.f1.docx]

**Supplementary Data**

**Title: Optimized Multi-Scale Entropy Model Based on Resting-State fMRI for Appraising Cognitive Performance in Healthy Elderly**

**Journal:** **Computational and Mathematical Methods in Medicine**

**Authors:** Fan Yang,^1,2^ Fuyi Zhang,^1,2^ Abdelkader Nasreddine Belkacem,^3^ Chong Xie,^1,2^ Ying Wang,^4^ Shenghua Chen,^1,2^ Zekun Yang,^1,2^ Zibo Song,^1,2^ Manling Ge,^1,2^ Chao Chen^4^

^1^ State Key Laboratory of Reliability and Intelligence of Electrical Equipment, Hebei University of Technology, Tianjin, 300130, China.
^2^ Hebei Province Key Laboratory of Electromagnetic Field and Electrical Apparatus Reliability, Hebei University of Technology, Tianjin, 300130, China.
^3^ Department of Computer and Network Engineering, College of Information Technology, United Arab Emirates University, Al Ain, 15551, UAE.
^4^ Key Laboratory of Complex System Control Theory and Application, Tianjin University of Technology, Tianjin, 300384, China.

Correspondence should be addressed to Shenghua Chen; chenshenghua@hebut.edu.cn and Chao Chen; chao_chen@emial.tjut.edu.cn

1. **Major depressive disorder (MDD)**

**Participants.** 56 patients diagnosed with major depressive disorder and 56 normal controls participated in this investigation. Patients were recruited from Chongqing Medical School and diagnosed by an experienced psychiatrist according to the Diagnostic and Statistical Manual of Mental Disorders-IV. Normal controls were recruited from the local community around Southwest University, Chongqing. The study was approved by the Southwest University Brain Imaging Center Institutional Review Board.

**Image Acquisition and Preprocessing.** A total of 242 volumes of resting-state functional magnetic resonance imaging (rfMRI) were obtained for each subject using an echo-plana imaging (EPI) sequence through a 3T Siemens Trio scanner (TR/TE = 2000/ 30ms, flip angle = 90°, acquisition matrix = 64×64, field of view=220×220mm^2^, axial slices=32, and thickness/gap = 3/1mm).

Functional data preprocessing was carried out using SPM8 (http://www.fil.ion.ucl.ac.uk/spm).  The entire process included removal of the first 10 volumes, slice timing correction, realignment to the first volume for head-motion correction, filtering (0.01-0.08 Hz), normalization to the EPI template with a resampling voxel size of 3x3x3 mm^3^, smoothing with a 6mm full-width at half-maximum Gaussian kernel. No subjects were excluded because all the head motions were less than 2mm or 2°.

We first employed the automated anatomical labeling (AAL) template to parcellate the brain into 90 regions of interest (ROIs). The time series were acquired on each ROI by averaging the signals of all voxels within that area and then linearly regressing out the influences of head motion and global signal.

**Result.** When *r* = 0.05~0.40, the entropy values of brain regions are invalid because there is no matching vector in the calculation process due to the small *r*. To ensure the accuracy of the analysis, the similarity *r* is selected in the range of 0.40~0.60 (step size is 0.02). According to Figure S1, the following optimization parameters can be obtained *m* = 1 and *r* = 0.48~0.54.


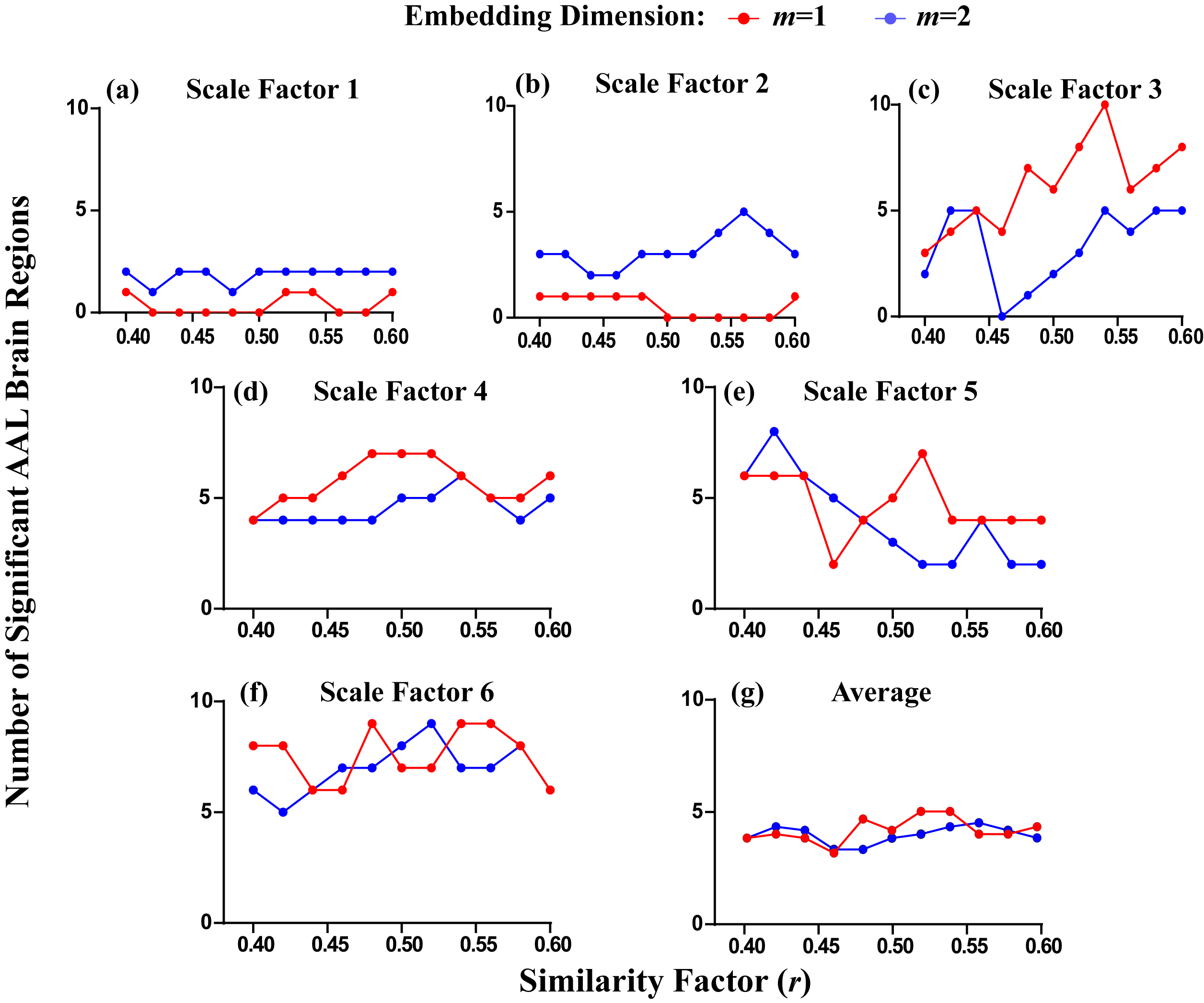


Figure S1: Optimization of embedding dimension *m* by the number of significant brain regions. (a)$\text{τ}$ = 1; (b)$\text{τ}$ = 2; (c) $\text{τ}$= 3; (d) $\text{τ}$= 4; (e) $\text{τ}$= 5; (f) $\text{τ}$= 6; (g) Average number of significant brain regions over the scale factor $\text{τ}$.

By setting *m* = 1 fixed and *r* = 0.48, 0.50, 0.52, and 0.54, respectively, the ROC curves of IPL.L at different $\text{τ}$ values are shown in Figure S2(a)–(d). The ROC curves around the reference lines displayed that the SOG.R cannot be a functional biomarker in Figure S2(e)–(h).


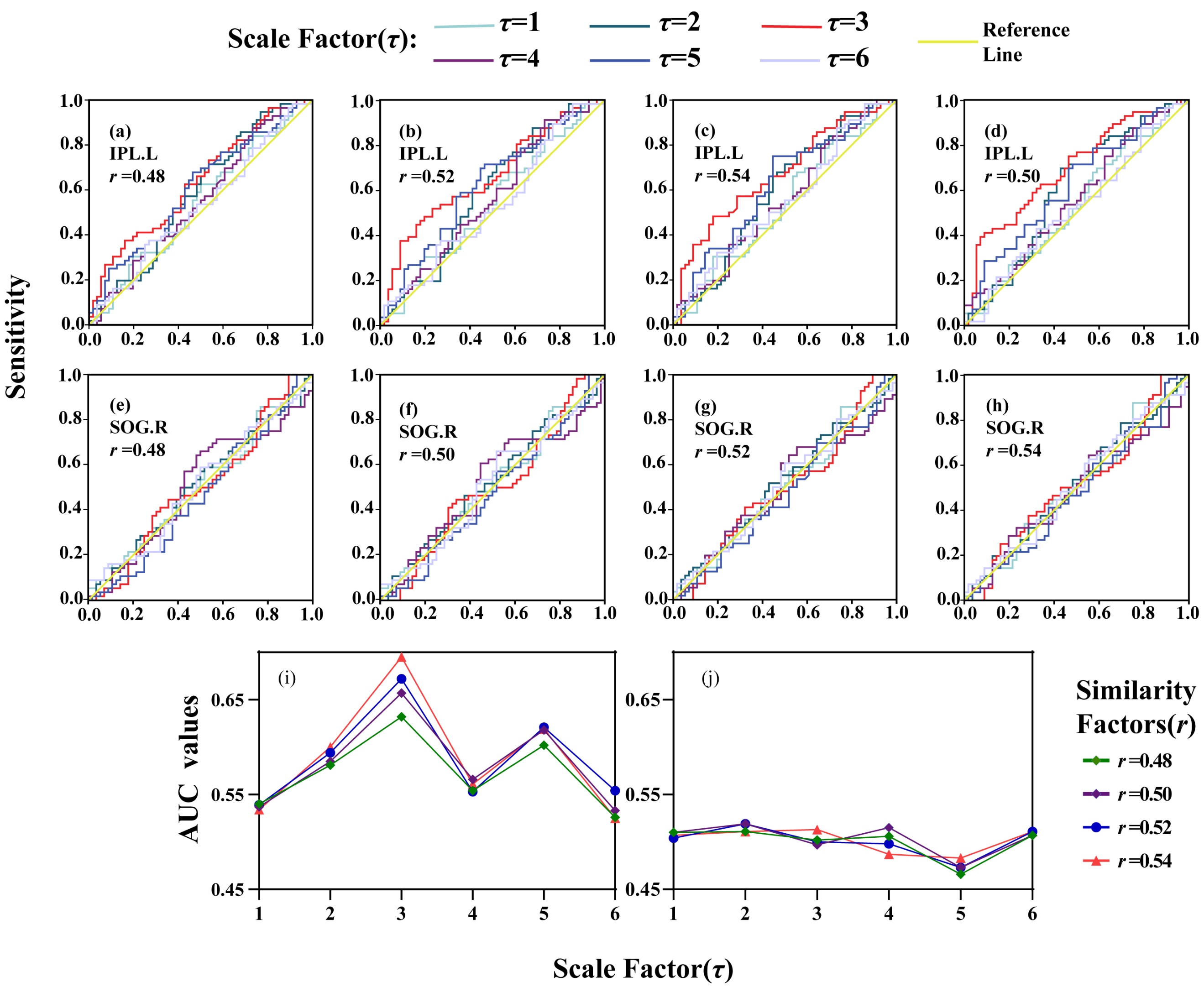


Figure S2: Optimization effects indicated by ROC curves and AUC values in a single brain region. (a)–(d) ROC curves of IPL.L with *r* = 0.48, 0.50, 0.52, and 0.54, respectively, where the character of all ROC curves beyond the reference lines indicates IPL.L to be a functional biomarker; (e)–(h) ROC curves of SOG.R with *r* = 0.48, 0.50, 0.52 and 0.54, respectively, where ROC curves around the reference lines suggest SOG.R was not a functional biomarker; (g) AUC values of IPL.L; (h) AUC values of SOG.R.

Furthermore, in Figure S2 (i)–(j), it was found that the AUC values of IPL.L were greater than those of SOG.R, and they were largest at $\text{τ}$ = 3, suggesting that $\text{τ}$ =3 was the optimized value. In particular, when$\text{τ}$ = 3, the AUC value of IPL.L was the largest at *r* = 0.54 as highlighted in Table S1, indicating that *r* = 0.54 was the optimized value.

Table S1: Effect of similarity factor *r* and scale factor $\text{τ}$ on sorting rate by AUC value of each brain region.

|  | r | 0.48 | | 0.50 | | 0.52 | | 0.54 | |
| --- | --- | --- | --- | --- | --- | --- | --- | --- | --- |
| $\text{τ}$ |  | IPL.L | SOG.R | IPL.L | SOG.R | IPL.L | SOG.R | IPL.L | SOG.R |
| 1 | | 0.540 | 0.510 | 0.537 | 0.510 | 0.539 | 0.504 | 0.534 | 0.507 |
| 2 | | 0.581 | 0.511 | 0.585 | 0.519 | 0.594 | 0.519 | 0.600 | 0.511 |
| 3 | | 0.632 | 0.502 | 0.657 | 0.497 | 0.672 | 0.500 | 0.695 | 0.513 |
| 4 | | 0.554 | 0.506 | 0.566 | 0.515 | 0.553 | 0.498 | 0.561 | 0.487 |
| 5 | | 0.602 | 0.466 | 0.618 | 0.473 | 0.621 | 0.473 | 0.619 | 0.483 |
| 6 | | 0.526 | 0.507 | 0.533 | 0.507 | 0.554 | 0.511 | 0.525 | 0.511 |

Taken together, the optimized parameters of entropy model were *m* = 1, *r* = 0.54, and$\text{τ}$ = 3. With the optimized MSE values of *m* = 1, *r* = 0.54, and $\text{τ}\text{ }$= 3, a total of nine AAL brain regions sensitive to MDD were obtained (*p* <0 .05), see Table S2. And their projections on the cortical surface are depicted in Figure S3.

Table S2: Significance (*p*-value) in a biomarker areas

| Brain Region | Significance (*p*-value) |
| --- | --- |
| IPL.L (AAL61) | 0.001 |
| AMYG.L (AAL41) | 0.002 |
| ORBsupmed. (AAL26) | 0.012 |
| PCUN.L (AAL67) | 0.019 |
| PCG.L (AAL35) | 0.024 |
| IFGtriang.L (AAL13) | 0.026 |
| ORBsup.L (AAL5) | 0.026 |
| SFGdor.R (AAL4) | 0.027 |
| HES.R (AAL80) | 0.028 |


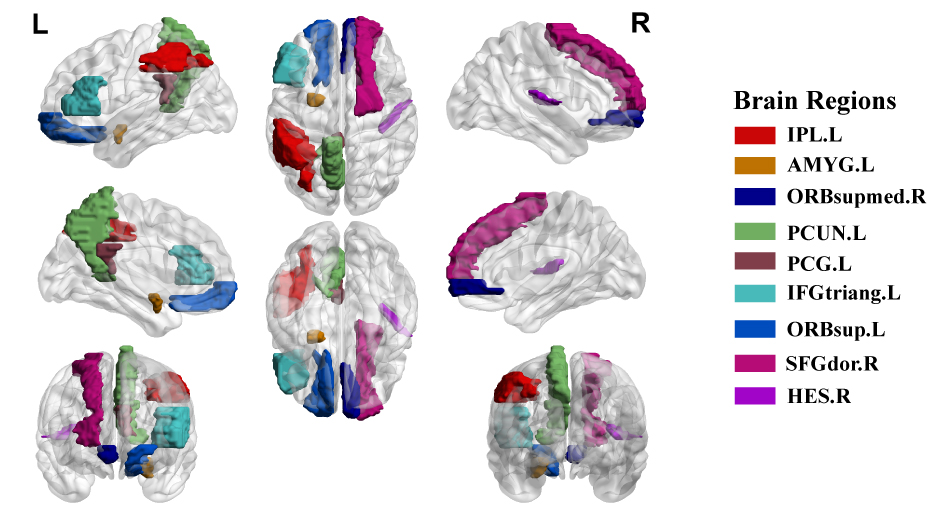


Figure S3: Landmark brain regions shown on a brain template using BrainNet Viewer.

Finally, the 10-fold cross validation was carried out in the probabilistic neural network (PNN), the max classification accuracy was 83.33%, and the average accuracy was 75.83%. (See Table S3).

Table S3: (MDD)Classification rate (CR) tested by 10-fold cross validation

| N | CR(%) | N | CR(%) | N | CR(%) |
| --- | --- | --- | --- | --- | --- |
| 1 | 75.00 | 6 | 83.33 |  |  |
| 2 | 83.33 | 7 | 66.67 |  |  |
| 3 | 83.33 | 8 | 83.33 | Average $\text{±}$ std | 75.83 $\text{±}$ 7.29 |
| 4 | 75.00 | 9 | 75.00 |  |  |
| 5 | 66.67 | 10 | 66.67 |  |  |

1. **Conventional FC model of Pearson correlation**

First, each subject got a 90×90 brain FC matrix and then averaged each row to form a 90×1 FC matrix. Then, 98 old people will get a 98×90 FC matrix. 9 brain regions with significant differences between groups were selected by the *t*-test used in this paper, and their FC values were input into the PNN model as feature vectors to classify and validate by the 10-fold cross validation. The classification results are shown in Table S4. Its average classification accuracy is 60.33%, which is worse than the classification accuracy of the optimized entropy model proposed in this paper. Because the entropy could reflect the dynamics in the non-linear system, it could be more suitable for the complicated brain system than the custom FC model of Pearson correlation.

Table S4: Classification rate (CR) tested by 10-fold cross validation in PNN

| N | CR(%) | N | CR(%) | N | CR(%) |
| --- | --- | --- | --- | --- | --- |
| 1 | 58.33 | 6 | 77.78 |  |  |
| 2 | 54.55 | 7 | 55.56 |  |  |
| 3 | 54.55 | 8 | 55.56 | Average $\text{±}$ std | 60.33$\text{±}$9.81 |
| 4 | 63.64 | 9 | 55.56 |  |  |
| 5 | 77.78 | 10 | 50.00 |  |  |

1. **Other different machine learning models**

Support Vector Machine (SVM) can classify data separated by non-linear and linear boundaries, originating from Vapnik's statistical learning theory. K-Nearest Neighbours (KNN) is a supervised learning approach that classifies data according to the majority of its neighbors. Based on previous work, we considered a range of K values between 1 and 30. The highest classification accuracy was obtained when K=11. Random Forest (RF) is one of the representatives of integrated learning algorithms, which has a better classification effect than individual models. The algorithm for building decision trees has ID3, C4.5, and CART. This paper uses the CART decision tree as RF basic classifier, and the sum of the decision tree is 500 lessons. First, extract N samples from the original data through the Bagging algorithm, so that each sample will generate a decision tree, and the generated decision tree does not need to be pruned. Thus, a forest with N decision trees is constructed. The classification accuracy of different machine learning is shown in Table S5.

Table S5: (MDD)Classification rate (CR) tested by 10-fold cross validation in different machine learning

| N | SVM | RF | KNN (K=11) | PNN |
| --- | --- | --- | --- | --- |
| 1 | 75.00 | 77.78 | 81.82 | 88.24 |
| 2 | 63.64 | 72.73 | 72.73 | 70.59 |
| 3 | 72.73 | 63.64 | 75.00 | 81.82 |
| 4 | 81.82 | 63.64 | 54.54 | 68.95 |
| 5 | 77.78 | 66.67 | 77.78 | 81.82 |
| 6 | 66.67 | 55.56 | 55.56 | 81.82 |
| 7 | 77.78 | 66.67 | 77.78 | 88.24 |
| 8 | 77.78 | 50.00 | 66.67 | 68.95 |
| 9 | 77.78 | 66.67 | 75.00 | 88.24 |
| 10 | 75.00 | 75.00 | 77.78 | 81.82 |
| Average $\text{±}$ std | 74.60$\text{±}$5.57 | 65.84$\text{±8.45}$ | 71.47$\text{±9.52}$ | 80.05$\text{±}$7.82 |
